# Rice plastidial NAD‐dependent malate dehydrogenase 1 negatively regulates salt stress response by reducing the vitamin B6 content

**DOI:** 10.1111/pbi.13184

**Published:** 2019-07-02

**Authors:** Nan Nan, Jie Wang, Yuejie Shi, Yangwen Qian, Long Jiang, Shuangzhan Huang, Yutong Liu, Ying Wu, Bao Liu, Zheng‐Yi Xu

**Affiliations:** ^1^ Key Laboratory of Molecular Epigenetics of the Ministry of Education (MOE) Northeast Normal University Changchun China; ^2^ Biogle Genome Editing Center Changzhou Jiangsu Province China; ^3^ School of Agronomy Jilin College of Agricultural Science & Technology Jilin China

**Keywords:** salt stress, malate dehydrogenase, pyridoxine, vitamin B6, reactive oxygen species, RNA‐sequencing, Rice

## Abstract

Salinity is an important environmental factor that adversely impacts crop growth and productivity. Malate dehydrogenases (MDHs) catalyse the reversible interconversion of malate and oxaloacetate using NAD(H)/NADP(H) as a cofactor and regulate plant development and abiotic stress tolerance. Vitamin B6 functions as an essential cofactor in enzymatic reactions involved in numerous cellular processes. However, the role of plastidial MDH in rice (*Oryza sativa*) in salt stress response by altering vitamin B6 content remains unknown. In this study, we identified a new loss‐of‐function *osmdh1* mutant displaying salt stress‐tolerant phenotype. The *OsMDH1* was expressed in different tissues of rice plants including leaf, leaf sheath, panicle, glume, bud, root and stem and was induced in the presence of NaCl. Transient expression of *OsMDH1‐GFP* in rice protoplasts showed that OsMDH1 localizes to chloroplast. Transgenic rice plants overexpressing *OsMDH1* (*OsMDH1OX*) displayed a salt stress‐sensitive phenotype. Liquid chromatography–mass spectrometry (LC‐MS) metabolic profiling revealed that the amount of pyridoxine was significantly reduced in *OsMDH1OX* lines compared with the *NIP* plants. Moreover, the pyridoxine content was higher in the *osmdh1* mutant and lower in *OsMDH1OX* plants than in the *NIP* plants under the salt stress, indicating that OsMDH1 negatively regulates salt stress‐induced pyridoxine accumulation. Furthermore, genome‐wide RNA‐sequencing (RNA‐seq) analysis indicated that ectopic expression of *OsMDH1* altered the expression level of genes encoding key enzymes of the vitamin B6 biosynthesis pathway, possibly reducing the level of pyridoxine. Together, our results establish a novel, negative regulatory role of *OsMDH1* in salt stress tolerance by affecting vitamin B6 content of rice tissues.

## Introduction

Salt stress is a major environmental factor affecting plant growth and development. Efforts to increase salt stress tolerance of crop plants would enable sustainable agriculture on marginal lands and improve crop yields. Salinity causes ionic stress (mainly because of sodium [Na^+^], chloride [Cl^‐^] and sulphate [${\text{SO}}_{4}^{2-}$ ] ions), osmotic stress and secondary stresses including nutritional imbalances and oxidative stress (Zhu, 2002). Salinity‐mediated oxidative stress reduces the availability of CO_2_ and consumption of NADPH by the Calvin cycle. When the level of ferredoxin is reduced during photosynthetic electron transfer, electrons are transferred from photosystem I (PS I) to oxygen to form superoxide radicals (${\text{O}}_{2}^{-}$) via the Mehler reaction, which initiates chain reactions that produce more oxygen radicals (Hsu and Kao, 2003). Reactive oxygen species (ROS) are continuously generated during normal metabolic processes in peroxisomes, mitochondria and cytoplasm, which destroy normal metabolism by damaging proteins, nucleic acids and lipids (McCord, 2000). Plants have evolved efficient ROS removal systems including ROS‐scavenging antioxidative enzymes and small nonenzymatic molecules such as polyphenolic compounds, carotenoids, anthocyanin, flavonoids, glutathione, ascorbate and α‐tocopherol.

Vitamin B6, which comprises pyridoxal, pyridoxine, pyridoxamine and their phosphorylated derivatives, is an essential cofactor of numerous metabolic enzymes involved in amino acid metabolism and antibiotic biosynthesis (Tambasco‐Studart *et al*., 2005). Moreover, vitamin B6 is a potent antioxidant, with particular ability to quench ROS, and plays a key role in biotic and abiotic stress responses (Chen and Xiong, 2005; Denslow *et al*., 2007; González *et al*., 2007; Tambasco‐Studart *et al*., 2005). Recently, *de novo* biosynthesis of vitamin B6 has been unravelled in plants (Burns *et al*., 2005; Ehrenshaft *et al*., 1999; Osmani *et al*., 1999; Raschle *et al*., 2005, 2007; Tambasco‐Studart *et al*., 2005). Pyridoxal phosphate synthase protein 1 (PDX1) and PDX2, which are essential for the biosynthesis of vitamin B6, form a complex that directly synthesizes the cofactor form of the vitamin, pyridoxal 5′‐phosphate, from ribose‐5‐phosphate, glyceraldehyde‐3‐phosphate and glutamine (Burns *et al*., 2005; Raschle *et al*., 2005). The *Arabidopsis thaliana* genome encodes three functional homologs of PDX1, AtPDX1.1, AtPDX1.2 and AtPDX1.3, and a single homolog of PDX2 (Raschke *et al*., 2011; Tambasco‐Studart *et al*., 2005). Arabidopsis loss‐of‐function *pdx1.1* and *pdx1.3* mutants are sensitive to photoinhibition and salt and osmotic stresses (Titiz *et al*., 2006). Moreover, vitamin B6 deficiency caused by loss‐of‐function mutation of *PDX1.3* reduces the antioxidant capacity of *pdx1.3* mutant plants (Havaux *et al*., 2009). All organisms are able to interconvert different vitamin B6 via the salvage pathway (Lum *et al*., 2002; Sang *et al*., 2007; Shi and Zhu, 2002). Three proteins in the salvage pathway have been identified in plants, namely, the PN/PL/PM kinase (SOS4 in Arabidopsis), pyridoxine/pyridoxamine 5′‐phosphate oxidase (PDX3 in Arabidopsis) and pyridoxal reductase (PLR1 in Arabidopsis) (Herrero *et al*., 2011; Lum *et al*., 2002; Sang *et al*., 2007; Shi and Zhu, 2002).

NAD(P)H and ATP are required for major energy‐consuming reactions such as the assimilation, biosynthesis, transport and regulation of carbon (C), nitrate (${\text{NO}}_{3}^{-}$) and ${\text{SO}}_{4}^{2-}$ during photoautotrophic and heterotrophic phases of plant growth under light and darkness (Selinski *et al*., 2014). Because the plasma membrane is impermeable to NADH and NADPH, plants employ specific translocators for the exchange of malate and oxaloacetate (OAA), which enables the indirect transport of reducing equivalents between different cellular compartments (Scheibe, 2004; Selinski *et al*., 2014). Thus, malate valves function as a powerful system for maintaining the ATP/NAD(P)H ratio required in various compartments (Scheibe, 2004). Malate dehydrogenase (MDH) isoforms play a key role in energy homeostasis in plant cells (Selinski *et al*., 2014). MDHs are oxidoreductases that catalyse the reversible interconversion of malate and OAA using NAD^+^ or NADP^+^ as a coenzyme, depending on the MDH isoform (Ocheretina and Scheibe, 1997). NAD‐MDH activities have been detected in cytosol and different subcellular organelles including chloroplasts, mitochondria and microbodies (Berkemeyer *et al*., 1998; Christine, 1992). The Arabidopsis genome encodes nine MDH isoforms including two plastidial, two mitochondrial, two peroxisomal and three cytosolic isoforms that do not harbour localization signals (Schreier *et al*., 2018). All nine MDH isoforms use NAD^+^ as a cofactor, except one of the plastidial isoforms, which uses NADP^+^ as the cofactor (Schreier *et al*., 2018). In Arabidopsis, mutants lacking the NAD^+^‐dependent plastidial MDH (*pdnad*‐*mdh*) are embryo‐lethal, and constitutive silencing of *pdNAD‐MDH* (*miR‐mdh‐1*) causes a pale green, dwarf phenotype. Intriguingly, both active and inactive forms of pdNAD‐MDH interact with a heteromeric AAA‐ATPase complex at the inner membrane of the chloroplast envelope and stabilize FtsH12 (Schreier *et al*., 2018). Recently, it has been reported that *FLO16* encoding a NAD‐dependent cytosolic MDH in rice plays a role in starch biosynthesis (Teng *et al*., 2019). The ATP content was reduced in the *flo16* mutant, leading to significant reduction in the activity of enzymes involved in starch biosynthesis (Teng *et al*., 2019). However, the function of plastidial MDHs in rice remains unknown.

In this study, we identified a loss‐of‐function *osmdh1* mutant, showing salt stress‐tolerant phenotypes. *OsMDH1OX* lines displayed salt stress‐sensitive phenotypes, concomitant with dramatically increased MDH activity, which was impaired in the *osmdh1* mutant under salt stress. Liquid chromatography–mass spectrometry (LC‐MS) metabolic analysis showed that the amount of pyridoxine was significantly reduced in *OsMDH1OX* plants compared with *NIP* plants. Moreover, the amount of salt stress‐induced pyridoxine was higher in the *osmdh1* mutant and lower in the *OsMDH1OX* lines than in the *NIP* plants. Furthermore, genome‐wide RNA‐seq analysis revealed that *OsMDH1* overexpression altered the expression of genes encoding key enzymes in the vitamin B6 biosynthesis pathway, leading to low pyridoxine levels. Our results reveal a novel role of *OsMDH1* in affecting the level of pyridoxine under salt stress.

## Results

### Loss‐of‐function *OsMDH1* mutants exhibit salt stress‐tolerant phenotypes

**Figure 1:**
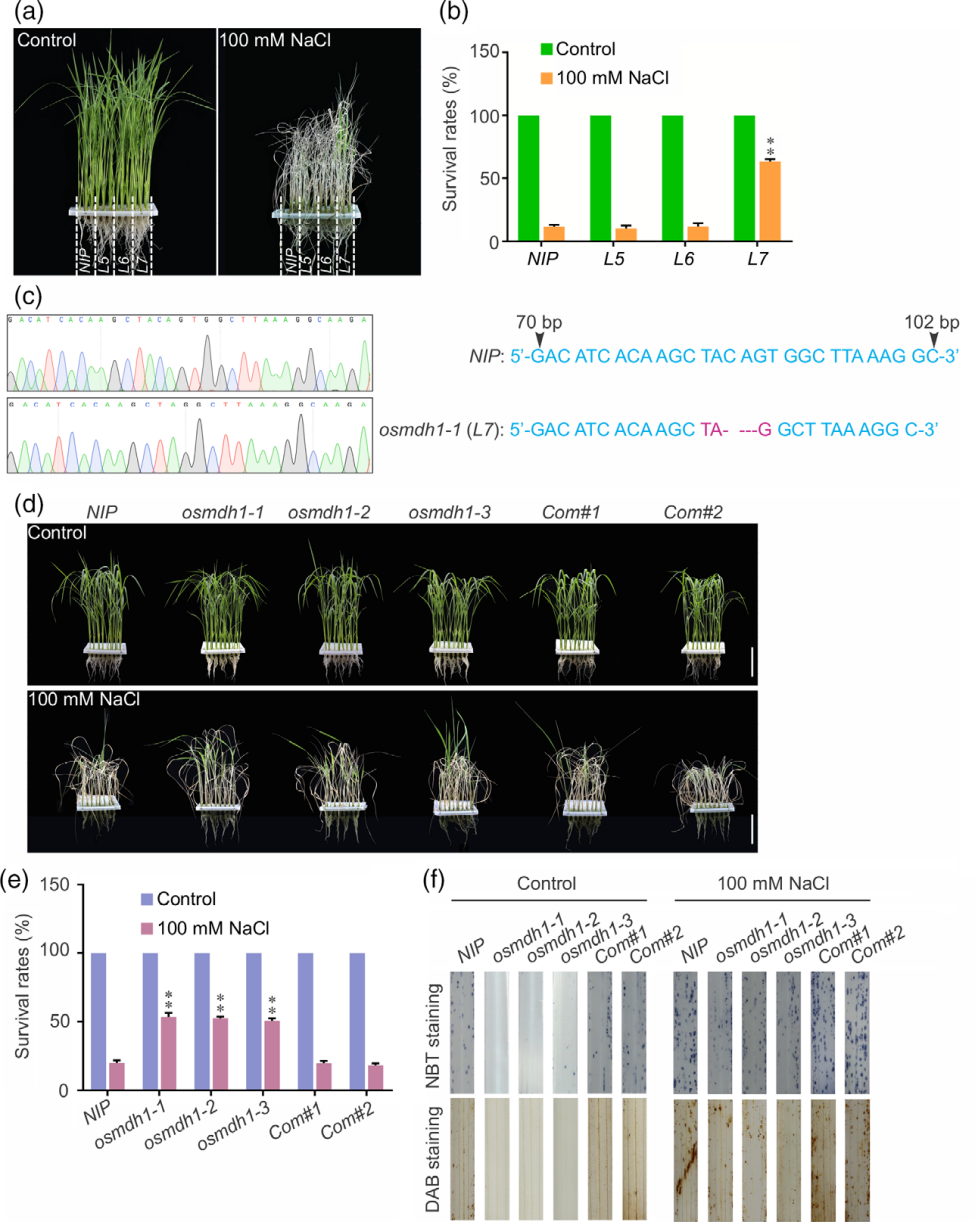
Loss‐of‐function *osmdh1* mutants exhibit salt tress‐tolerant phenotypes. (a) Isolation of salt stress‐responsive mutants from the CRISPR/Cas9 mutant pool RGKO‐ALL (mutant line 5, 6 and 7 (*L5*,* L6*, and *L7*) are different mutant lines obtained from the CRISPR/Cas9 mutant pool). *NIP* represents cultivar Nipponbare. Images were taken before 100 mm NaCl treatment and after recovery from the NaCl treatment. (b) The survival rates were measured before 100 mm NaCl treatment and after recovery from the NaCl treatment. Error bars indicate ± SD (*n *=* *3). Statistical analyses were performed by comparing *L5*,* L6* and *L7* mutants with *NIP* plants, respectively. **, *P‐*value <0.01 (Student's *t*‐test). (c) Sanger sequencing chromatography showing the mutation of *osmdh1‐1* (*L7*) including the deletion of C, A, G and T. (d) Phenotypes of *NIP* plants, three *osmdh1* mutants (*osmdh1‐1*,* osmdh1‐2* and *osmdh1‐3*) and two complementation lines (*Com#1* and *Com#2*) before 100 mm NaCl treatment and after recovery from the NaCl treatment. Bars, 4 cm. (e) The survival rates were measured before 100 mm NaCl treatment and after recovery from the NaCl treatment. Error bars indicate ± SD (*n *=* *3). Statistical analyses were performed by comparing three *osmdh1* mutants and two complementation lines with *NIP* plants, respectively. **, *P‐*value <0.01 (Student's *t*‐test). (f) Accumulation of ROS in the leaves under the normal condition and the salt stress condition. Nitrotetrazolium blue chloride (NBT) and diaminobenzidine (DAB) staining were used to assess the accumulation of ${\text{O}}_{2}^{-}$ and H_2_O_2_, respectively. Seedlings were treated with or without 100 mm NaCl for 12 h before staining. Each treatment was analysed using 20 plants. Three biological repeats were performed.

In a forward genetic screening of a genome‐scale mutagenesis library of rice (*Oryza sativa* L. var. Nipponbare) CRISPR/Cas9 (clustered regularly interspaced short palindromic repeats‐associated nuclease 9) mutant pool RGKO‐ALL (a genome‐scale mutagenesis library of rice) (Lu *et al*., 2017), we identified a mutant line 7 (*L7*) showing increased tolerance to 100 mm NaCl treatment compared with the wild type (*NIP*) (Figure 1a,b). Information obtained from the barcoded next‐generation sequencing (NGS) data and Sanger sequencing analysis (Lu *et al*., 2017) was combined, which revealed a 4 bp deletion located 84 bp downstream of the ATG start codon of the gene encoding MDH1 (LOC_Os01g61380), resulting in the generation of a premature stop codon in *L7* mutant (*osmdh1‐1*) (Figure 1c). The rice genome encodes approximately 10 MDHs, of which OsMDH1 contains highly conserved NAD^+^‐binding and proton acceptor sites, which are required for its activity (Figure S1). To confirm whether the loss‐of‐function mutation of *OsMDH1* increased salt tolerance, we generated two independent mutants, *osmdh1‐2* and *osmdh1‐3*, using the CRISPR/Cas9 system. Specific guide RNA (gRNA) target sites for *OsMDH1* were cloned into the CRISPR/Cas9 vector, in which *Cas9* was driven by the *UBQ10* promoter (Ma *et al*., 2015). The vectors were then transformed into *NIP* plants, and homozygous *osmdh1‐2* and *osmdh1‐3* mutant lines were identified via Sanger sequencing (Figure S2a). In the *osmdh1‐2* mutant, 1 bp deletion was found 647 bp downstream of the ATG, resulting in a frame shift mutation and consequently a premature stop codon before the core catalytic domain (Figure S2a). In the *osmdh1‐3* mutant, 1 bp insertion was detected 742 bp downstream of the ATG, causing a frame shift (Figure S2a). To exclude the potential confounding effect of the *Cas9* gene *per se*, we isolated *osmdh1‐1*,* osmdh1‐2* and *osmdh1‐3* mutants by screening for nonhygromycin resistance (Figure S2b). *Cas9*‐free *osmdh1‐1*,* osmdh1‐2* and *osmdh1‐3* mutants were used in this study. The *osmdh1‐2* and *osmdh1‐3* mutants displayed salt stress‐tolerant phenotypes similar to *osmdh1‐1* (Figure 1d,e). To further confirm whether the loss‐of‐function mutation of *OsMDH1* was responsible for salt stress tolerance, we generated complementation lines by transfecting the *osmdh1‐1* mutant with a construct expressing *OsMDH1* cDNA fused to *GFP* under the control of the *OsMDH1* promoter (*OsMDH1*_*pro*_ *:OsMDH1‐GFP*). Two independent complementation lines (*Com#1* and *Com#2*) were chosen for further analysis, and the level of OsMHD1‐GFP protein in these lines was detected using Western blot analysis (Figure S2c). Both *Com#1* and *Com#2* lines showed similar survival rates compared with *NIP* plants under salt stress (Figure 1d,e). The reduced rate of photosynthesis under salt stress increases the production of ROS including hydrogen peroxide (H_2_O_2_) and ${\text{O}}_{2}^{-}$ (Sharma *et al*., 2012). Staining of leaves with diaminobenzidine (DAB) and nitrotetrazolium blue chloride (NBT) revealed that *osmdh1* mutants accumulated less ROS than *NIP* plants under normal conditions and under salt stress (Figure 1f). Of notes, *osmdh1* mutants displayed a late flowering phenotype compared with *NIP* plants under normal conditions (Figure S2d,e). Taken together, these results indicate that OsMDH1 participates in salt stress tolerance.

### *OsMDH1OX* plants exhibit salt stress‐sensitive phenotypes

**Figure 2:**
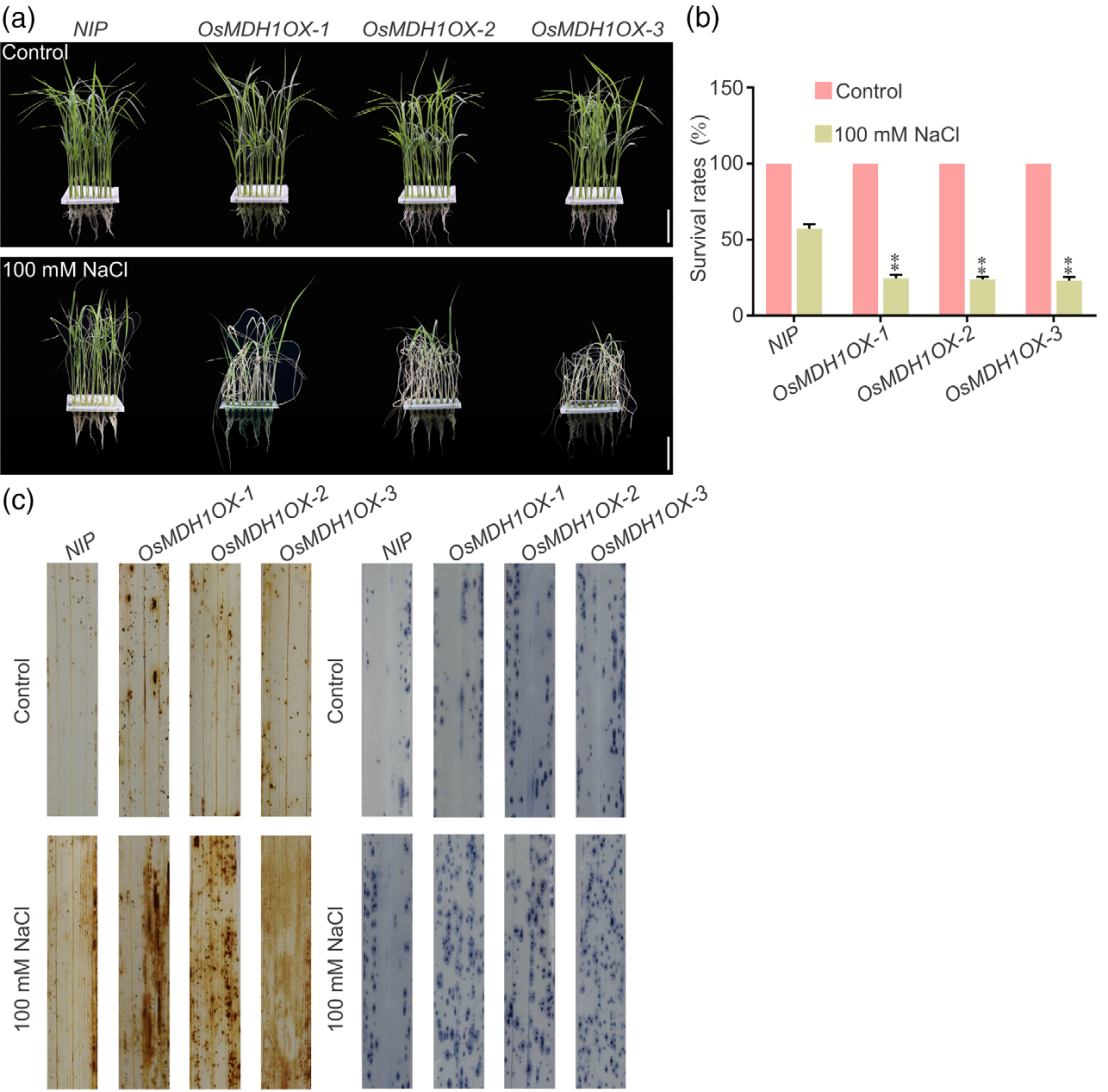
Transgenic rice plants overexpressing *OsMDH1* (*OsMDH1OX*) exhibit salt tress‐sensitive phenotypes. (a) Phenotypes of *NIP* plants and *OsMDH1OX* lines (*OsMDH1OX‐1*,* OsMDH1OX‐2* and *OsMDH1OX‐3*) before the 100 mm NaCl treatment and after recovery from the NaCl treatment. Bars, 4 cm. (b) The survival rates were measured before 100 mm NaCl treatment and after recovery from the NaCl treatment. Error bars indicate ± SD (*n *=* *3). Statistical analyses were performed by comparing *OsMDH1OX* lines with *NIP* plants. **, *P‐*value <0.01 (Student's *t*‐test). (c) Accumulation of ROS in the leaves under the normal condition and the salt stress condition. NBT and DAB staining was used to assess the accumulation of ${\text{O}}_{2}^{-}$ and H_2_O_2_, respectively. Seedlings were treated with or without 100 mm NaCl for 12 h before staining. Each treatment was analysed using 20 plants. Three biological repeats were performed.

To further investigate the role of *OsMDH1* in salt stress tolerance, we generated three independent *OsMDH1OX* lines (*OsMDH1OX‐1*,* OsMDH1OX‐2* and *OsMDH1OX‐3*). Real‐time quantitative PCR (RT‐qPCR) analysis revealed that transcript levels of *OsMDH1* were dramatically increased in all three *OsMDH1OX* lines (Figure S3a). The *OsMDH1OX* lines displayed an early flowering phenotype compared with *NIP* plants under normal conditions (Figure S3b,c). Under the salt stress, *OsMDH1OX* lines showed salt stress‐sensitive phenotypes compared with *NIP* plants, and the survival rate of *OsMDH1OX* lines was lower than that of *NIP* plants (Figure 2a,b). Additionally, we detected higher levels of ROS in *OsMDH1OX* lines than in *NIP* plants (Figure 2c).

### Tissue‐specific expression patterns of *OsMDH1* and subcellular localization of OsMDH1

**Figure 3:**
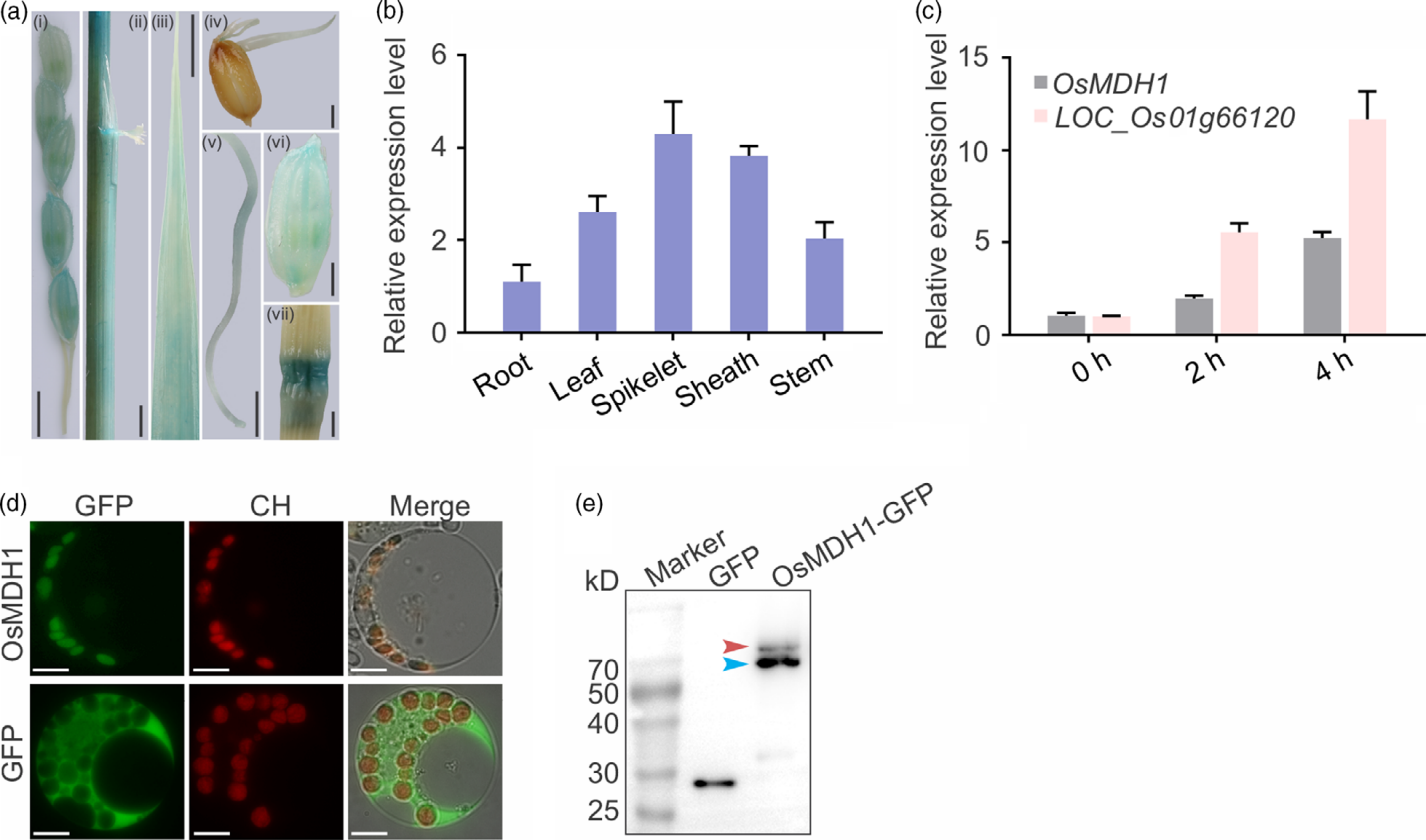
Tissue‐specific expression patterns of *OsMDH1* and subcellular localization of OsMDH1. (a) β‐glucuronidase (GUS) staining for spatial and temporal expression patterns of *OsMDH1*. Transgenic plants expressing the β*‐glucuronidase* (*GUS*) gene under the control of the *OsMDH1* promoter (*OsMDH1*_*pro*_ *:GUS*) at different developmental stages were stained with 5‐bromo‐4‐chloro‐3‐indolyl β‐D‐glucuronide (X‐Gluc). GUS signals were examined in panicle (i), leaf sheath (ii), leaf (iii), bud (iv), root (v), glume (vi) and stem node tissues (vii). Bars, 5 mm (i, ii, iii, v); 2 mm (iv, vi, vii). (b) Using RT‐qPCR detected *GUS* expression levels to confirm GUS staining results at different tissues and developmental stages. Error bars indicate ± SD (*n *=* *3). (c) RT‐qPCR analysis of *OsMDH1* under 100 mm NaCl treatment for 0, 2 and 4 h. Gene (*LOC_Os01g66120*) was as a positive control under the salt stress. *OsACT1* was used as an internal control. Error bars indicate ± SD (*n *=* *3). (d) Subcellular localization of OsMDH1‐GFP. Protoplasts from *NIP* plants were transformed with *OsMDH1‐GFP*. Free GFP served as a control. The signals were observed under a fluorescence microscope. GFP, green fluorescent protein; CH, chloroplast autofluorescence. Cell images were also taken under bright field as a control. Bars, 10 μm. (e) Western blot analysis of GFP and OsMDH1‐GFP was performed using a monoclonal mouse anti‐GFP antibody. The band with higher molecular weight marked with the red arrow is the premature form of OsMDH1 with transit peptide, and blue arrow indicates the molecular weight of mature OsMDH1 after transit peptide cleavage.

To examine the spatial and temporal expression patterns of *OsMDH1*, we generated transgenic plants expressing the β*‐glucuronidase* (*GUS*) gene under the control of the *OsMDH1* promoter (*OsMDH1*_*pro*_ *:GUS*) and examined the promoter activity of *OsMDH1* in different tissues at distinct developmental stages. GUS signals were detected in leaf, leaf sheath, panicle, glume, bud, root and stem tissues (Figure 3a). We also confirmed these results using RT‐qPCR (Figure 3b). Additionally, *OsMDH1* transcripts were rapidly induced under 100 mm NaCl treatment (Figure 3c); the gene (*LOC_Os01g66120*), a NaCl‐responsive marker, was used as a positive control in this experiment (Figure 3c).

To examine the subcellular localization of OsMDH1, we cloned the *GFP* gene at the 3′ end of the *OsMDH1* coding sequence to generate the *OsMDH1‐GFP* fusion. The *OsMDH1‐GFP* construct was transfected into rice protoplasts. GFP signals colocalized with chloroplast autofluorescence, indicating that OsMDH1 localizes to the chloroplast (Figure 3d). Western blot analysis using anti‐GFP antibody revealed two bands (Figure 3e). We speculated that the higher molecular weight band represented a premature form of OsMDH1‐GFP harbouring a transit peptide, while the lower molecular weight band represented the mature OsMDH1‐GFP protein localized to chloroplast after transit peptide cleavage. We also confirmed the existence of the transit peptide using the ChloroP 1.1 Server bioinformatics tool (Table S1) (Emanuelsson *et al*., 1999).

### Analysis of OsMDH1 biochemical activity

**Figure 4:**
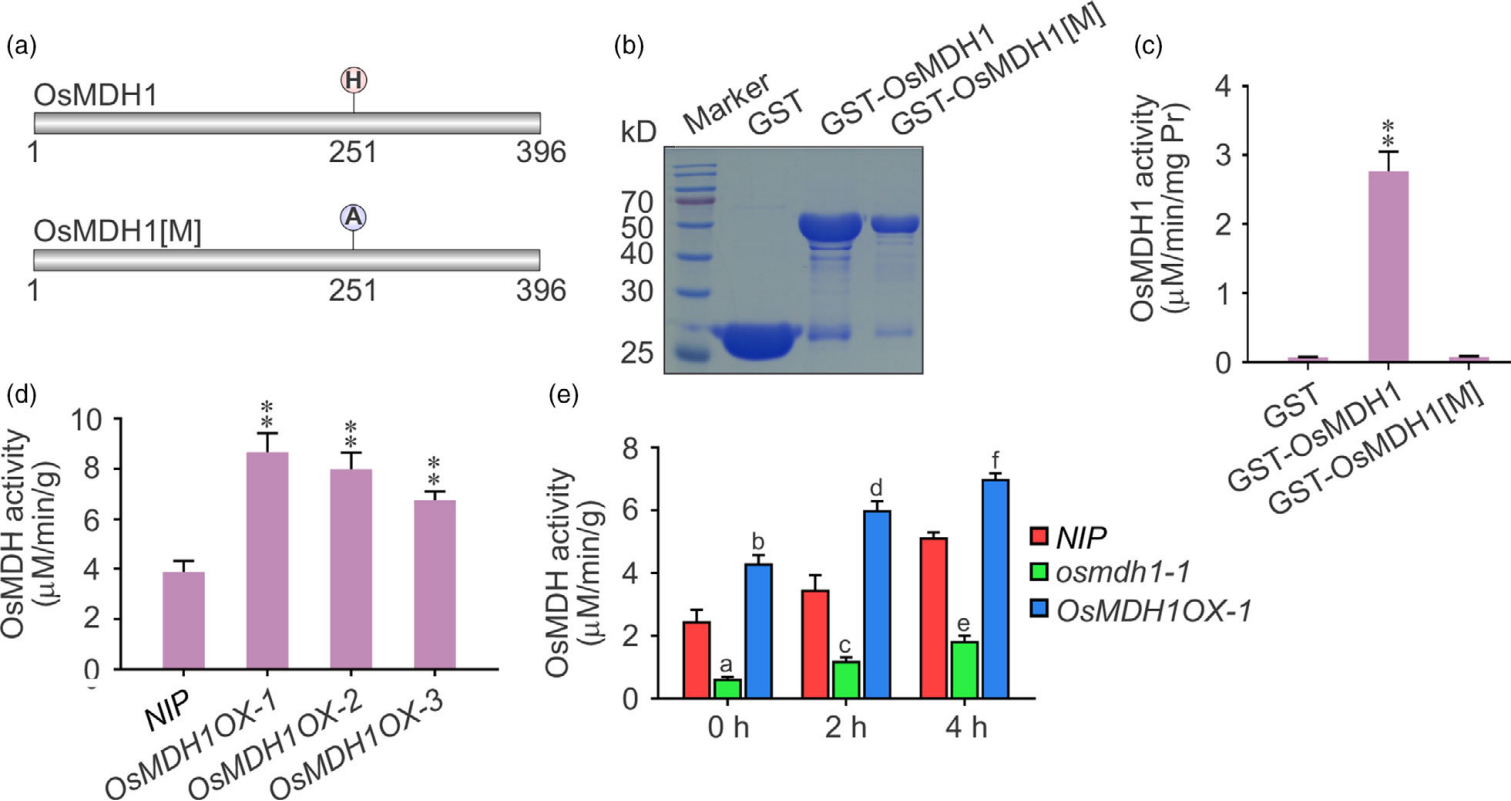
Analysis of OsMDH1 biochemical activity *in vitro* and *in vivo*. (a) Putative core catalytic site of OsMDH1 using the Universal Protein Resource (UniProt, http://www.uniprot.org). Histidine was the core catalytic site of OsMDH1 and was substituted by alanine in catalytically inactive form of OsMDH1[M]. (b) Coomassie blue‐stained gel showing levels of recombinant GST proteins (GST, GST‐OsMDH1 and GST‐OsMDH1[M]) used in detecting malate dehydrogenase activity. (c) The malate dehydrogenase activities of GST, GST‐OsMDH1 and GST‐OsMDH1[M] were determined by the NADH oxidation. Error bars indicate ± SD (*n *=* *3). Statistical analyses were performed by comparing GST‐OsMDH1 with GST and GST‐OsMDH1[M], respectively. **, *P‐*value <0.01 (Student's *t*‐test). (d) Examination of the malate dehydrogenase activities of chloroplast proteins from *NIP* and *OsMDH1OX* plants under normal conditions. Error bars indicate ± SD (*n *=* *3). Statistical analyses were performed by comparing *OsMDH1OX* lines with *NIP* plants. **, *P‐*value <0.01 (Student's *t*‐test). (e) Examination of the malate dehydrogenase activities of chloroplast proteins from *NIP*,* osmdh1‐1* and *OsMDH1OX‐1* plants under 100 mm NaCl treatment for 0, 2 and 4 h. Error bars indicate ± SD (*n *=* *3). Statistical analyses were performed by comparing *osmdh1‐1* and *OsMDH1OX‐1* with *NIP* plants, respectively, at the same NaCl treatment time. (Student's *t*‐test, a = 2.12*10^−6^, b = 5.27*10^−5^, c = 3.05*10^−5^, d = 2.62*10^−5^, e = 1.08*10^−7^, and f = 9.62*10^−6^).

To examine the biochemical activity of OsMDH1 *in vitro*, we expressed an N‐terminal fusion of OsMDH1 with glutathione S‐transferase (GST) in *Escherichia coli*. We also tested the GST fusion of the catalytically inactive form of OsMDH1[M], in which histidine located at the core catalytic domain was substituted with alanine (Figure 4a). The recombinant proteins were purified (Figure 4b) and incubated with OAA and NADH. We detected that the activity of NAD‐MDH was dramatically increased after introducing OAA and NADH in GST‐OsMDH1, whereas the activities of GST and GST‐OsMDH1[M] showed no changes (Figure 4c). To test the biochemical activity of OsMDH1 *in vivo*, we purified chloroplast proteins from *NIP* plants and *OsMDH1OX* lines and incubated chloroplast proteins with OAA and NADH. The activity of NAD‐MDH was dramatically increased in *OsMDH1OX* lines compared with *NIP* plants (Figure 4d). Taken together, these results indicate that OsMDH1 is a plastid‐localized, NAD‐dependent enzyme. Since salt stress rapidly induces the expression of *OsMDH1*, we further examined the NAD‐MDH activity under salt stress using chloroplast proteins purified from *NIP*,* osmdh1‐1* and *OsMDH1OX‐1* plants. The results showed that plastidial NAD‐MDH activity was dramatically increased in *NIP* plants under salt stress but only marginally altered in *osmdh1‐1* mutants (Figure 4e). Additionally, plastidial NAD‐MDH activity increased more quickly in the *OsMDH1OX‐1* line than in *NIP* plants under salt stress (Figure 4e). These results indicate that salt stress up‐regulates the expression of *OsMDH1*, thus inducing plastidial OsMDH1 activity.

### Ectopic expression of *OsMDH1* alters metabolic profiles

**Figure 5:**
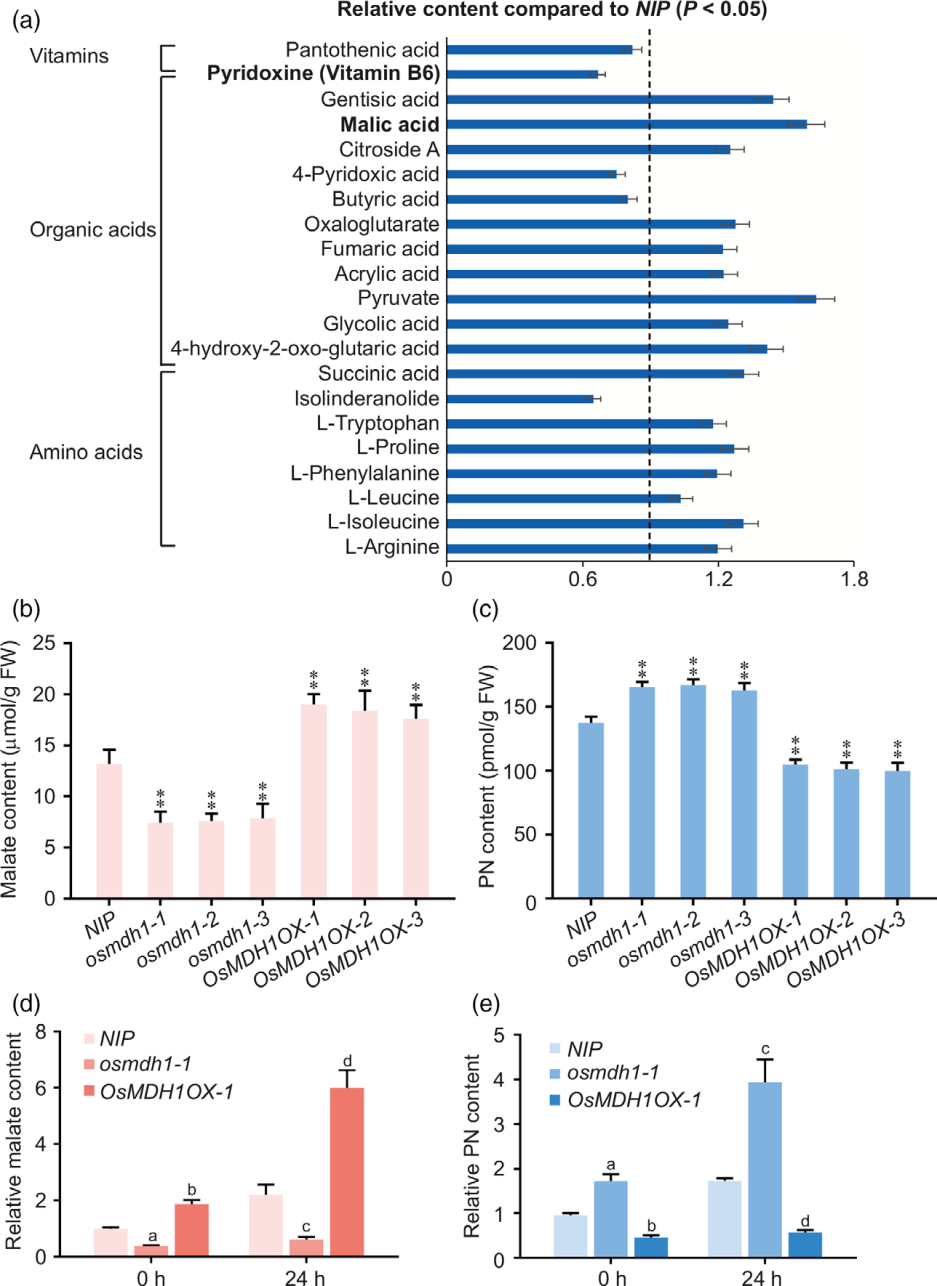
Ectopic expression of *OsMDH1* causes metabolic alterations in different pathways. (a) Metabolites including vitamins, organic acids and amino acids were selected from differential metabolites identified between *OsMDH1OX‐1* and *NIP* plants under normal conditions. Error bars indicate ± SD (*n *=* *10). (b, c) Malate and pyridoxine contents in *NIP*,* osmdh1* and *OsMDH1OX* plants were measured under normal conditions. Error bars indicate ± SD (*n *=* *10). Statistical analyses were performed by comparing *osmdh1* mutants and *OsMDH1OX* lines with *NIP* plants, respectively. **, *P‐*value <0.01 (Student's *t*‐test). PN, pyridoxine. (d) Malate contents in *NIP*,* osmdh1‐1* and *OsMDH1OX‐1* plants were measured under 100 mm NaCl treatment for 0 and 24 h. Relative malate content of *osmdh1‐1* and *OsMDH1OX‐1* plants was normalized by malate content in *NIP* plants. Error bars indicate ± SD (*n *=* *10). Statistical analyses were performed by comparing *osmdh1‐1* and *OsMDH1OX‐1* with *NIP* plants, respectively. (Student's *t*‐test, a = 4.82*10^−3^, b = 3.56*10^−4^, c = 7.42 *10^−5^, and d = 7.52*10^−6^). (e) Pyridoxine contents in *NIP*,* osmdh1‐1* and *OsMDH1OX‐1* plants were measured under 100 mm NaCl treatment for 0 and 24 h. Relative pyridoxine content of *osmdh1‐1* and *OsMDH1OX‐1* plants was normalized by pyridoxine content in *NIP* plants. Error bars indicate ± SD (*n *=* *10). Statistical analyses were performed by comparing *osmdh1‐1* and *OsMDH1OX‐1* with *NIP* plants, respectively. (Student's *t*‐test, a = 6.82*10^−4^, b = 7.92*10^−3^, c = 5.83 *10^−6^, and d = 9.16*10^−6^). PN, pyridoxine.

To determine the metabolic consequences of the ectopic expression of *OsMDH1*, we compared the metabolic profiles of *NIP* and *OsMDH1OX‐1* plants using LC‐MS under normal conditions (Przyborowska *et al*., 2004). The metabolome was analysed using three methods: orthogonal projections to latent structures discriminant analysis (OPLS‐DA), partial least square discriminant analysis (PLS‐DA) and principal components analysis (PCA). A clear separation was observed between *NIP* and *OsMDH1OX‐1* plants (Figure S4). Compared with *NIP* plants, levels of 144 metabolites were higher and 41 metabolites were lower in *OsMDH1OX‐1* plants (Table S2). The differentially accumulated metabolites were mapped to KEGG (Kyoto Encyclopedia of Genes and Genomes) ID using MetaboAnalyst online software (Xia and Wishart, 2016). The results showed that ‘flavone and flavonol biosynthesis’ and ‘tyrosine metabolism’ pathways were significantly enriched (*P *<* *0.05; Table S3). Additionally, malic acid content was dramatically increased in *OsMDH1OX‐1* plants compared with *NIP* plants, which is consistent with the sharp increase in NAD‐MDH activity in *OsMDH1OX‐1* plants compared with *NIP* plants (Figure 5a). We further confirmed this result in three independent *OsMDH1OX* lines and three *osmdh1* mutant lines. Compared with *NIP* plants, malate contents of *OsMDH1OX* lines were significantly increased, while those of *osmdh1* mutants were dramatically reduced (Figure 5b). Intriguingly, we found that the amount of pyridoxine was lower in *OsMDH1OX* lines and significantly higher in *osmdh1* mutants than in *NIP* plants (Figure 5a,c). We further examined the effect of salt stress on malate and pyridoxine contents of plants. Under the salt stress, both malate and pyridoxine contents were increased in *NIP* plants, whereas *osmdh1‐1* mutants showed a marginal change in malate level and a dramatic increase in pyridoxine content (Figure 5d,e). Additionally, *OsMDH1OX‐1* plants contained more malate and less pyridoxine than *NIP* plants under salt stress (Figure 5d,e). These results indicate that ectopic expression of *OsMDH1* induces metabolic alterations in different pathways.

### Pyridoxine improves salt tolerance of *OsMDH1OX* plants

**Figure 6:**
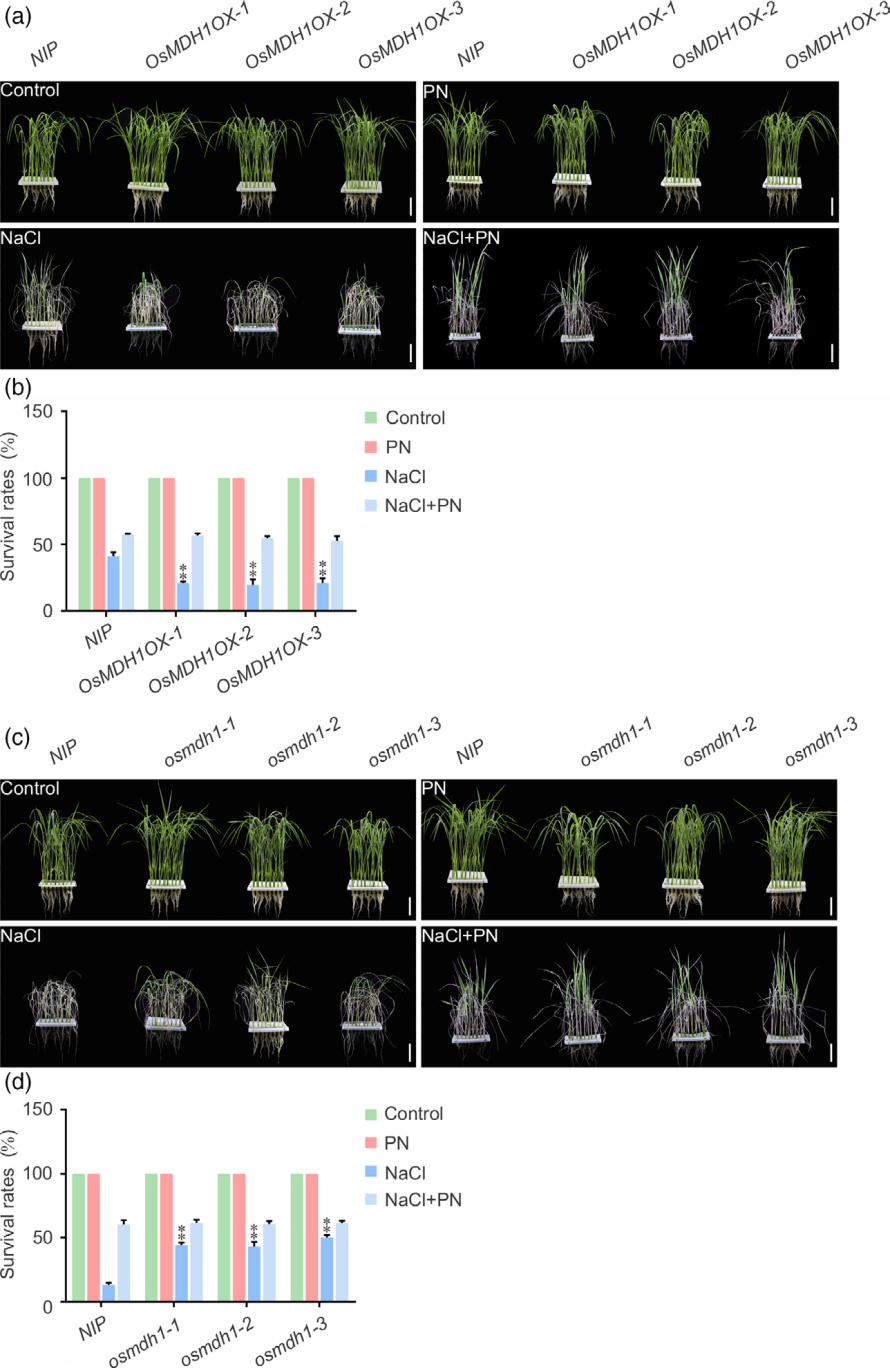
Pyridoxine improves the salt stress tolerance of *OsMDH1OX* plants. Phenotypes of *OsMDH1OX* lines (a, b) and *osmdh1* mutants (c, d) grown on the culture solution with 10 μm PN, 100 mm NaCl or 10 μm PN and 100 mm NaCl. PN, pyridoxine. Images were taken, and the survival rates were measured before the treatment and after the recovery from the treatment. Bars, 4 cm. Error bars indicate ± SD (*n *=* *3). Statistical analyses were performed by comparing *OsMDH1OX* lines and *osmdh1* mutants with *NIP* plants, respectively. **, *P‐*value <0.01 (Student's *t*‐test).

To determine whether the salt stress‐sensitive phenotype of *OsMDH1OX* was caused by the reduction in the amount of pyridoxine, we examined whether the addition of pyridoxine to the culture solution could rescue the salt stress‐sensitive phenotype of *OsMDH1OX* lines. Under normal conditions, the addition of pyridoxine to the culture solution caused no phenotypic alterations in *OsMDH1OX* lines compared with *NIP* plants. However, in the presence of NaCl, addition of 10 μm pyridoxine to the culture solution rescued the salt stress‐sensitive phenotype of *OsMDH1OX* lines (Figure 6a,b). We also examined the effect of 10 μm pyridoxine on loss‐of‐function *osmdh1* mutant lines in the presence or absence of 100 mm NaCl. Under normal conditions, we observed no differences between *NIP* and *osmdh1* plants upon the addition of pyridoxine. By contrast, in the presence of 100 mm NaCl and no pyridoxine, the *osmdh1* mutant showed salt stress‐tolerant phenotypes, whereas *NIP* plants were sensitive to salt stress. However, after the addition of pyridoxine to the culture solution, *NIP* plants and *osmdh1* mutant lines showed similar phenotypes (Figure 6c,d). Taken together, these results indicate that plant pyridoxine levels determined by OsMDH1 play an important role in the salt stress response.

### Ectopic expression of *OsMDH1* alters gene expression

**Figure 7:**
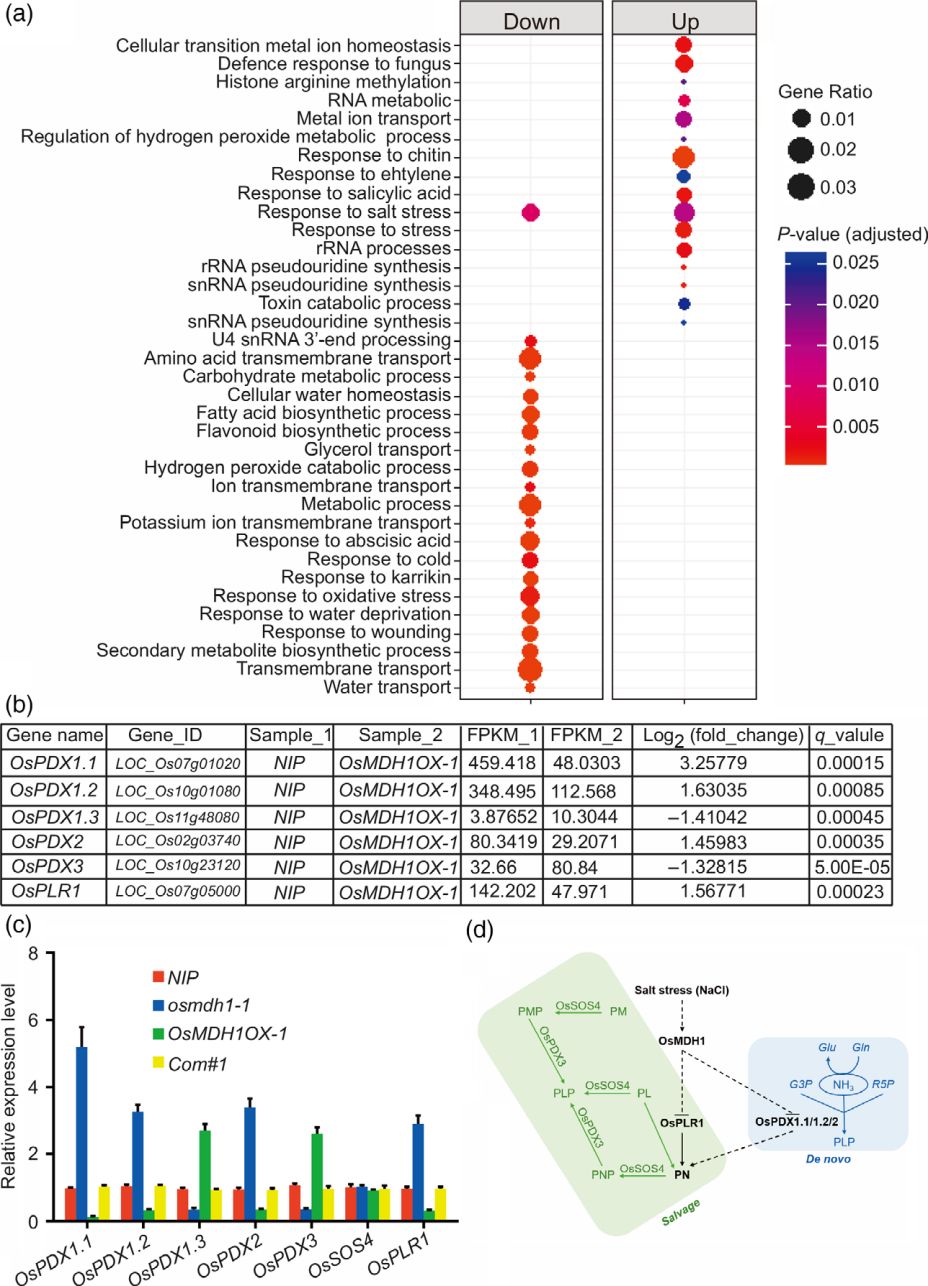
Ectopic expression of *OsMDH1* alters genes expression. (a) Gene ontology (GO) analyses were performed to categorize functions of differentially expressed genes (DEGs). (b) RNA‐sequencing results of genes encoding key enzymes involved in vitamin B6 biosynthesis including gene name, gene identity (gene_ID), FPKM (Fragments Per Kilobase Million) values, log_2_(fold changes) as well as *q*‐values. (c) RT‐qPCR analysis of genes encoding key enzymes involved in vitamin B6 biosynthesis. *OsACT1* was used as an internal control. Error bars indicate ± SD (*n *=* *3). (d) Under the salt stress, transcriptional level of *OsMDH1* is increased, which indirectly leads to transcriptional levels of *OsPLR1*,* OsPDX1.1*,* OsPDX1.2* and *OsPDX2* decreased. Subsequently, the down‐regulated genes can reduce pyridoxine content directly or indirectly. Pathways of the biosynthesis of vitamin B6 in plants including *de novo* pathway (*De novo*) and salvage pathway (*Salvage*). Pyridoxal 5′‐phosphate (PLP) is produced *de novo* from ribose 5‐phosphate (R5P), glyceraldehyde 3‐phosphate (G3P) and glutamine (Gln) via the action of PDX1 and PDX2. Enzymes of the salvage pathway can interconvert the vitameric forms, that is pyridoxine (PN), pyridoxal (PL), pyridoxamine (PM) or their phosphorylated derivatives (PNP, PLP and PMP, respectively) as depicted. The PN/PM oxidase (PDX3), PN/PL/PM kinase (SOS4) and pyridoxal reductase (PLR1) have been identified in plants.

To determine the effect of *OsMDH1* overexpression on the transcriptome, we performed RNA‐seq analysis under normal conditions (Table S4). A total of 1059 genes were up‐regulated and 2516 genes were down‐regulated in *OsMDH1OX* plants compared with *NIP* plants (Table S5). Gene enrichment analysis revealed that gene ontology (GO) terms including ‘response to salt stress’, ‘response to cold stress’, ‘response to water deprivation’, ‘response to abscisic acid’ and ‘cellular water homeostasis’ were significantly enriched among the down‐regulated genes, whereas GO terms including ‘defense response to fungus’, ‘response to chitin’ and ‘response to ethylene’ were enriched among the up‐regulated genes (Figure 7a; Table S6). Using RT‐qPCR, we further examined the expression levels of genes encoding key enzymes involved in vitamin B6 biosynthesis including *OsPDX1.1*,* OsPDX1.2*,* OsPDX1.3*,* OsPDX2*,* OsPDX3*,* OsSOS4* and *OsPLR1*. Consistent with RNA‐seq results (Figure 7b), we found that the expression of *OsPDX1.1*,* OsPDX1.2*,* OsPDX2* and *OsPLR1* was substantially reduced, while expression of *OsPDX1.3* and *OsPDX3* was increased in *OsMDH1OX* lines compared with *NIP* plants (Figure 7c). Notably, the expression of *OsSOS4* did not show noticeable alterations between *OsMDH1OX* lines and *NIP* plants (Figure 7c). These results indicate that ectopic expression of *OsMDH1* alters the expression of genes encoding key regulators of vitamin B6 biosynthesis, which possibly reduces the pyridoxine content. Thus, we conclude that, under salt stress, overexpression of *OsMDH1* indirectly impacts the expression of genes, such as *OsPLR1*,* OsPDX1.1*,* OsPDX1.2* and *OsPDX2*, which reduces the pyridoxine content (Figure 7d).

## Discussion

In this study, we screened a genome‐wide mutagenesis library of rice CRISPR/Cas9 mutant pool (Lu *et al*., 2017) and identified a salt stress‐tolerant loss‐of‐function mutant, *osmdh1*. We showed that *OsMDH1* is expressed in leaf, leaf sheath, panicle, glume, bud, root and stem tissues of rice plants and is rapidly induced by NaCl treatment. The activity of OsMDH1 was dramatically higher in *OsMDH1OX* lines and significantly impaired in the *osmdh1‐1* mutant. In plants, it has been reported that MDH is important in providing malate for C4 metabolism, pH balance, stomatal and pulvinal movement, respiration, β‐oxidation of fatty acids and legume root nodule functioning (Miller *et al*., 1998). Since ectopic expression of *OsMDH1* leads to changes of metabolic profiles, we guess the alteration of metabolome in *OsMDH1*‐overexpression lines might cause the genes expression change. RNA‐seq analysis of *OsMDH1OX* transgenic plants revealed that GO terms including ‘response to salt stress’, ‘response to cold stress’, ‘response to water deprivation’, ‘response to abscisic acid’ and ‘cellular water homeostasis’ were significantly enriched among the down‐regulated genes, while ‘defense response to fungus’, ‘response to chitin’ and ‘response to ethylene’ were enriched among the up‐regulated genes. Thus, it is likely that OsMDH1 impacts the expression of genes in abiotic and biotic stress responses. In apple (*Malus domestica*), the cytosolic *MDH* gene (*MdcyMDH*) is induced by mild cold and salt stresses, and transgenic apple plants overexpressing *MydcyMDH* exhibit improved cold and salt tolerance compared with wild‐type plants (Wang *et al*., 2016). Under salt and cold stresses, the reductive activity of *MydcyMDH* overexpression lines was significantly higher than that of wild‐type plants (Wang *et al*., 2016). Although the reductive activities of OsMDH1 and MydcyMDH are increased under salt stress, plants overexpressing the corresponding genes display opposite phenotypes; lines overexpressing *OsMDH1* are sensitive to salt stress, whereas those overexpressing *MydcyMDH* lines are tolerant to salt stress. One of the possible explanations for this difference is that MDHs located in different subcellular compartments have different contributions to salt stress tolerance. It has been reported that plastidial malate dehydrogenases mostly contribute to generate malate and NAD by catalysing oxaloacetate using NADH as a cofactor (Scheibe, 2004)**.** After exportation from the chloroplast, malate can work as a source for NADH in the cytosol and feeds mitochondrial ATP production (Scheibe, 2004). Previously, Arabidopsis pdNAD‐MDH protein has been shown to play an important role in plastid development during embryogenesis (Beeler *et al*., 2014), and embryos of the *pdnad‐mdh* knockout mutant display arrested development during the globular‐to‐heart transition stage (Schreier *et al*., 2018). The embryo‐lethal phenotype of the *pdnad‐mdh* mutant is not dependent on enzyme activity of pdNAD‐MDH (Schreier *et al*., 2018). The majority of the pdNAD‐MDH proteins localize to the stroma and chloroplast envelope, while a small amount of the proteins associate with the thylakoid membranes (Ferro *et al*., 2010). The pdNAD‐MDH protein interacts with members of a proposed large AAA protease complex, comprising FtsH12 and FtsHi subunits and Ycf2, localized to the inner chloroplast membrane (Schreier *et al*., 2018). Intriguingly, NAD(H) and NADP(H) levels did not change in *pdNAD‐MDH* knock‐down mutant (*miR*‐*mdh*‐*1*) compared with wild‐type plants (Beeler *et al*., 2014). It is possible that OsMDH1 also participates in chloroplast development; however, whether and how salt stress‐responsive phenotype of OsMDH1 connected to chloroplast biogenesis remains to be investigated. Further studies should be performed by introducing the catalytically inactive form of OsMDH1 (OsMDH1[M]) into the *osmdh1* mutant background, to observe whether OsMDH1[M] is able to complement the salt stress‐tolerant phenotype of *osmdh1*.

In this study, *OsMDH1OX* transgenic lines showed an early flowering phenotype, indicating that OsMDH1 is involved in the regulation of flowering time in rice. Previously, it has been reported that salt stress delays flowering in Arabidopsis by reducing the transcript levels for *CO* and *FT* (Li *et al*., 2007). Exogenous application of gibberellin (GA) causes delayed or inhibited transition from vegetative growth to reproductive development (Li *et al*., 2007). In addition, the CONSTANS (CO)/FLOWERING LOCUS T (FT) module may also play a role in mediating the effects of salt on flowering (Li *et al*., 2007). In our RNA‐seq analysis, we detected that the expression of *OsCO3*, a negative regulator in flowering, was decreased in *OsMDH1OX‐1* compared with *NIP* (Table S5). Since the expression of *OsMDH1* was increased under the treatment of NaCl, we deduce that increased *OsMDH1* transcript level might lead to reduced expression of *OsCO3*, thereby causing early flowering in *OsMDH1OX* lines. Recently, a floury endosperm mutant in rice, *flo16*, was characterized and found to display defective starch grain formation (Teng *et al*., 2019). *FLO16* encodes a NAD‐dependent cytosolic MDH. The ATP content was reduced in the *flo16* mutant, leading to significant reduction in the activity of enzymes involved in starch biosynthesis (Teng *et al*., 2019). Thus, it is likely that *OsMDHs* located in different subcellular compartments play different physiological roles during plant development and growth. Similar functions of MDHs in plant growth and development have been investigated in other plant species. In Arabidopsis, mitochondrial MDH (mMDH) and pdMDH play crucial roles during embryogenesis; mMDH is also involved in photorespiration (Beeler *et al*., 2014; Sew *et al*., 2016). Additionally, Arabidopsis peroxisomal MDHs (PMDH1 and PMDH2) have function in plant growth (Pracharoenwattana *et al*., 2007). In tomato (*Solanum lycopersicum*), MDH regulates starch biosynthesis in the amyloplast (Centeno *et al*., 2011).

Vitamin B6 comprises six related compounds including pyridoxal, pyridoxine, pyridoxamine and the corresponding 5′ phosphorylated esters. Vitamin B6 is also thought to function as an antioxidant because the level of vitamin B6 is positively correlated with oxidative stress responses in both animals and plants (Hellmann and Mooney, 2010). According to the present study and a previous publication (Huang *et al*., 2013), pyridoxine content is most likely induced under NaCl treatment, indicating that vitamin B6 biosynthesis is one of the several important mechanisms that regulate ROS detoxification. This idea is further supported by our observations that ROS accumulation, determined using DAB or NBT staining, was impaired in the *osmdh1* mutant but enhanced in *OsMDH1OX* lines under normal conditions and salt stress. These results indicate that *OsMDH1* plays a key role in NaCl‐induced ROS detoxification in rice. We also found that OsMDH1 influenced NaCl‐induced pyridoxine accumulation, possibly by impacting the expression of *OsPLR1*,* OsPDX1.1*,* OsPDX1.2* and *OsPDX2*. In Arabidopsis, *AtPLR1* encodes a pyridoxal reductase involved in the vitamin B6 salvage pathway (Herrero *et al*., 2011). In rice, one homolog of AtPLR1 (OsPLR1) was identified, which showed 50.4% amino acid sequence similarity with AtPLR1. The *atplr1* loss‐of‐function mutant showed no noticeable phenotypes during plant development, except the salt stress‐sensitive phenotype (Herrero *et al*., 2011). Thus, it is possible that reduction in the expression of *OsPLR1* in the *OsMDH1OX* lines leads to the salt stress‐sensitive phenotype. The *AtPDX1* gene encodes a PLP synthase, while *AtPDX2* encodes a PLP glutaminase (Raschke *et al*., 2011). Ectopic expression of *AtPDX2* increases the level of vitamin B6 in shoots and desiccated seeds (Li, 2014). In the present study, the expression of *OsPDX2* was significantly reduced in *OsMDH1OX* lines compared with *NIP* plants; thus, it is possible that the reduction in *OsPDX2* expression impacts the homeostasis of vitamin B6 in *OsMDH1OX* lines. *AtSOS4* encodes a pyridoxal kinase, which is involved in pyridoxal 5′‐phosphate biosynthesis (Shi *et al*., 2002). The *atsos4* loss‐of‐function mutant is salt stress‐sensitive, and pyridoxine treatment significantly rescues the *atsos4* mutant phenotype, indicating that pyridoxine kinase converts pyridoxine to pyridoxine 5′‐phosphate, which is subsequently converted to pyridoxal 5′‐phosphate through the action of pyridoxine 5′‐phosphate oxidase (Shi *et al*., 2002). In *OsMDH1OX* lines, the expression of the *AtSOS4* homolog (*OsSOS4*) was not altered, indicating that low pyridoxine content was not caused by the reduction in pyridoxine kinase activity. In Arabidopsis, active uptake of vitamin B6 from external sources via the AtPUP1 transporter, which retrieves pyridoxine and pyridoxal exuded in the guttation fluid, has been reported previously (Szydlowski *et al*., 2013); however, whether and how the vitamin B6 transporter affects pyridoxine and pyridoxal contents remains unknown. Further studies are needed to investigate the exact mechanisms of the biosynthesis and transport of vitamin B6 under salt stress.

## Experimental procedures

### Plant growth and salt stress treatment conditions

Whole genome‐scale mutagenesis library of rice CRISPR/Cas9 mutant pool RGKO‐ALL was obtained from Biogle Genome Editing Center (Lu *et al*., 2017). Seeds from plants with different genotypes including wild‐type (*NIP*), *osmdh1* mutant lines and the transgenic lines were sterilized for 30 min with sodium hypochlorite solution and, subsequently, were washed three times with sterile distilled water. The seeds were soaked in water at 37 °C for 3 days. Seedlings were grown hydroponically in Yoshida's culture solution (Yoshida, 1976) and cultured in a growth chamber with the temperature at 28 °C/25 °C (day/night) under the 14‐h light/10‐h dark photoperiod (approximately 200 μm photons m^−2^ s^−1^). Four‐week‐old seedlings were subjected to salt stress (100 mm NaCl) for 5 or 7 days, respectively. After the treatment, the rice seedlings were transferred to NaCl‐free hydroponic culture solution for the recovery. Survival rates were measured after 3 days recovery, and survival rates were shown as percentages of alive seedlings. Plants not showing green shoots were regarded as dead seedlings. For testing the flowering phenotype, *NIP*,* osmdh1* and *OsMDH1OX* lines were grown in the soil with at least 10 plants in each genotype.

### Plasmid construction and generation of transgenic plants

Gene‐specific primers, OsMDH1‐F/R, were used to isolate *OsMDH1* cDNA from a cDNA library by PCR. To generate the *pCsV1300*‐*OsMDH1* construct, full‐length *OsMDH1* was amplified and cloned into the *pCsV1300* vector using the *Xba*I and *Bam*HI sites (Xu *et al*., 2012). To generate *OsMDH1‐GFP* construct for the rice protoplast transfection, *OsMDH1* cDNA was inserted into *326‐GFP* using *Xba*I and *Bam*HI sites (Jin *et al*., 2001). To generate *OsMDH1*_*pro*_ *:GUS* construct, 2.98‐kb fragment upstream of *OsMDH1* was amplified by PCR using primer sets, OsMDH1p‐F/R, and was inserted into the binary vector *pCAMBIA3301* using *Eco*RI and *Nco*I sites (Kim *et al*., 2013). To generate *GST‐OsMDH1* construct, full‐length *OsMDH1* cDNA fragment was amplified by PCR using primer sets, GST‐OsMDH1‐F/R, and catalytically inactive form of *OsMDH1* (*OsMDH1[M]*) was amplified by overlapping PCR using primer sets, GST‐OsMDH1‐F/R as well as OsMDH1[M]‐F/R, and were inserted into pGEX‐4T‐1 vector (Ahmad *et al*., 2019) using *Bam*HI and *Eco*RI sites, respectively. To knockout *OsMDH1* gene, two *OsMDH1* CRISPR/Cas9 constructs were designed using the *pYLsgRNA‐OsU6a* and *pYLCRISPR/Cas9Pubi‐H* plasmids according to the method described previously (Ma *et al*., 2015). CRISPR/Cas9 targeted sequences were designed in the following website (http://skl.scau.edu.cn/). Constructs were transformed into *NIP* plants by the Agrobacterium‐mediated transformation method (Hiei *et al*., 1994). PCR and Sanger sequencing were used to examine the mutation sequences. T2 seeds were screened with hygromycin (Liu *et al*., 2018). All primers are listed in Table S7.

### Tissue‐specific expression test using GUS staining and examination of subcellular localization

GUS staining experiment was performed using transgenic plants expressing the β*‐glucuronidase* (*GUS*) gene under the control of the *OsMDH1* promoter (*OsMDH1*_*pro*_ *:GUS*) as previously described (Jefferson *et al*., 1987). Rice protoplasts obtained from three‐week‐old seedlings (grown under 12‐h light and 12‐h dark conditions) were used for examining the subcellular localization of OsMDH1‐GFP (Zhang *et al*., 2011). The fluorescence microscope (Olympus BX53, Japan) was used to detect GFP and chloroplast autofluorescence signals. Western blot analysis was performed using a monoclonal mouse anti‐GFP antibody (Transgen Biotech, Cat. HT801‐01).

### NBT and DAB staining methods

The nitrotetrazolium blue chloride (NBT) and diaminobenzidine (DAB) staining assays were performed according to the method as previously described with a slight modification (Wu *et al*., 2017). Four‐week‐old seedlings were treated without or with 100 mm NaCl for 12 h. To detect ${\text{O}}_{2}^{-}$, the leaves of plants were vacuum infiltrated for 30 min and then stained for 12 h with 0.05% NBT (w/v) and 10 mm NaN_3_ in 10 mm potassium phosphate buffer (pH = 7.8). To detect H_2_O_2_, the leaves of plants were vacuum infiltrated for 1 h and then stained with 0.1% DAB for 24 h (pH = 5.8). Subsequently, leaves were incubated in the destaining buffer (ethanol:lactic acid:glycerol = 3:1:1) (Zhang *et al*., 2018).

### Protein purification

Different constructs expressing GST, GST‐OsMDH1 or GST‐OsMDH1[M] were transformed to *E. coli* BL21 cell line. Protein were purified as previously described (Xu *et al*., 2013).

### Chloroplast isolation

Chloroplasts were isolated from 1 g of leaves of four‐week‐old seedlings as described previously (van Wijk *et al*., 2007). The intactness of the purified chloroplast fraction was over 80%–90% as judged by the ferricyanide test. Protein amounts were quantified using Protein Assay Dye Reagent Concentrate (Bio‐Rad, Cat. 5000006).

### Analysis of OsMDH1 biochemical activity

Malate dehydrogenase activity of purified proteins including GST, GST‐OsMDH1, GST‐OsMDH1[M] and proteins extracted from chloroplasts was measured using Malate Dehydrogenase Assay Kit (Sigma, Cat. MAK196). Briefly, after initiating the reaction by adding MDH substrate, spectrophotometric change at 340 nm was monitored automatically at 40‐s intervals for 5 min. Activities were calculated according to the NADH standard curve after each measurement (Berkemeyer *et al*., 1998).

### Profiling of metabolites by liquid chromatography–mass spectrometry (LC‐MS)

Nearly 50 mg of leaves of four‐week‐old seedlings from *NIP* and *OsMDH1OX‐1* plants was incubated with 500 μL methanol with ten biological replications. The mixture was homogenized for 1 min at 70 Hz and mixed by vortex mixer for 30 s. Then, the mixture was centrifuged at 18 000 g 4 °C for 15 min. Two hundred micro litre supernatant was transferred to sampler vials and detected. An in‐house quality control (QC) was prepared by mixing equal amount of each sample. Agilent 1290 Infinity II UHPLC system coupled to an Agilent 6545 UHD and Accurate‐Mass Q‐TOF/MS was used for LC‐MS analysis (Ebert *et al*., 2010; Gabay *et al*., 2019).

### Metabolome analyses

The acquired MS data from GC‐MS were converted into the common data format (.mzdata) by Agilent MassHunter Qualitative Analysis version B.08.00 software (Agilent Technologies, Palo Alto, California, USA). Using the R software platform (https://www.r-project.org/, Lucent Technologies, New Providence, NJ), the XCMS was applied for data pretreatment such as nonlinear retention time alignment, peak discrimination, filtering, alignment, matching and identification. Subsequently, visualization matrices containing m/z and RT pair, peak area as well as sample names were obtained. Then, multivariate analyses including PCA, PLS‐DA and OPLS‐DA were conducted. The differential metabolites were screened out by combining VIP (Variable Importance in the Projection) value of OPLS‐DA model (VIP >=  1) with *P*‐value lower than 0.05 (Student's *t*‐test). Then, the differential metabolites were mapped to KEGG ID using MetaboAnalyst online software (https://www.metaboanalyst.ca/). Pathway analysis was conducted, and the model organism selected was *Oryza sativa* L. ssp. *japonica*. Pathways that have *P*‐value lower than 0.05 were picked out (Fiehn *et al*., 2000).

### Determination of pyridoxine and malate contents

The leaves samples of four‐week‐old seedlings were taken and immediately frozen in liquid nitrogen and stored at −80 °C until further analysis. Extraction was performed by rapid grinding of tissue in liquid nitrogen and immediate addition of the appropriate extraction buffer. Malate was measured by enzymatic analysis (Nunes‐Nesi *et al*., 2007). Pyridoxine was measured by HPLC as described previously (Szydlowski *et al*., 2013).

### RNA extraction and RT‐qPCR analysis

Total RNA was extracted with Trizol (Invitrogen, Cat. AM1912). Two microgram RNA were was used to prepare first‐strand cDNA using TransScript One‐Step gDNA Removal and cDNA Synthesis SuperMix (Transgen Biotech, Cat. AU311‐02). RT‐qPCR analysis was performed using the THUNDERBIRD SYBR qPCR Mix Without Rox reagent (TOYOBO, Cat. QPS‐20(‐)). Each sample was normalized against *OsACT1* control, and fold change relative to wild type was calculated according to the 2^‐△△CT^ method (Livak and Schmittgen, 2001). All primers are listed in Table S7.

### Bioinformatics analyses of RNA‐seq data

Total RNAs were extracted from *NIP* and *OsMDH1OX* plants and were subsequently used for transcriptome analysis using the Illumina HiSeq2500 platform (Illumina, San Diego, CA) with three biological replications. The Agilent 2100 Bioanalyzer (Agilent Technologies, Waldbronn, Germany) was used to determine the quality and concentration of RNA. Sequencing was performed in paired‐end mode with a read length of 150 nucleotides. Next, low‐quality (< Q20) reads were excluded from raw data using FASTX‐Toolkit v.0.0.13 (http://hannonlab.cshl.edu/fastx_tool-kit/). The clean reads were mapped to rice reference genome MSU7.0 using HISAT2 v.2.1.0 (https://ccb.jhu.edu-/software/hisat2/index.shtml) with default parameters (Kim *et al*., 2015). Gene quantification was performed using Cufflinks (http://cole-trapnell-lab.github.io/cufflinks/) with genomic annotation. The differentially expressed genes were filtered according to the fold change (|log_2_FC| >1) and an adjusted *P*‐value (<0.05), calculated with Cuffdiff (a subpackage of Cufflinks) (Yu *et al*., 2016, 2018). The gene ontology (GO) grouping of differentially expressed genes (DEGs) was performed by hypergeometric distribution in R v.3.1.0 (https://www.r-project.org/, Lucent Technologies), with an adjusted *P*‐value <0.05 as a cut‐off to determine significantly enriched GO terms.

## Accession number

Data generated in this study are deposited in NCBI Sequence Read Archive (accession number PRJNA528686).

## Author contributions

Z.‐Y.X. and N.N. devised and supervised the project. N.N. performed most of experiments and analysed data. J.W. and Y.J.S. generated transgenic plants. Y.W.Q. provided mutant lines. L.J., S.Z.H., Y.T.L. and Y.W. performed molecular cloning and some physiological assays. Z.‐Y.X. and B.L. wrote the manuscript. All authors reviewed, revised and approved the manuscript.

## Conflict of interest

The authors have no conflict of interest to declare.

## Supporting information


**Figure S1** Phylogenetic relationship of plastidial NAD‐MDH in monocot plants and catalytic sites prediction of OsMDH1.

**Figure S2** Phenotypes of the *osmdh1* mutants.

**Figure S3** Phenotypes of the *OsMDH1OX* lines.

**Figure S4** OPLS‐DA, PLS‐DA and PCA loading plots for the discrimination between *NIP vs OsMDH1OX‐1* plants under normal conditions.

**Table S1** Chloroplast transit peptide cleavage site prediction of OsMDH1 using ChloroP.
**Table S2** Differential metabolites identified comparing *OsMDH1OX‐1* with *NIP* plants under normal conditions.
**Table S3** Metabolic pathways of differential metabolites identified comparing *OsMDH1OX‐1* with *NIP* plants under normal conditions.
**Table S4** Reads numbers and data size of RNA‐Seq data.
**Table S5** Differentially expressed genes comparing *OsMDH1OX* with *NIP* plants under normal conditions.
**Table S6** Enriched GO terms of differentially expressed genes comparing *OsMDH1OX* with *NIP* plants under normal conditions.
**Table S7** Primer sequences used in different experiments.
