# Supplementary figures and images for: Rice plastidial NAD‐dependent malate dehydrogenase 1 negatively regulates salt stress response by reducing the vitamin B6 content
Source: Plant Biotechnol J. 2019 Jul 2;18(1):172–84. doi: 10.1111/pbi.13184 (PMC6920159; doi:10.1111/pbi.13184)

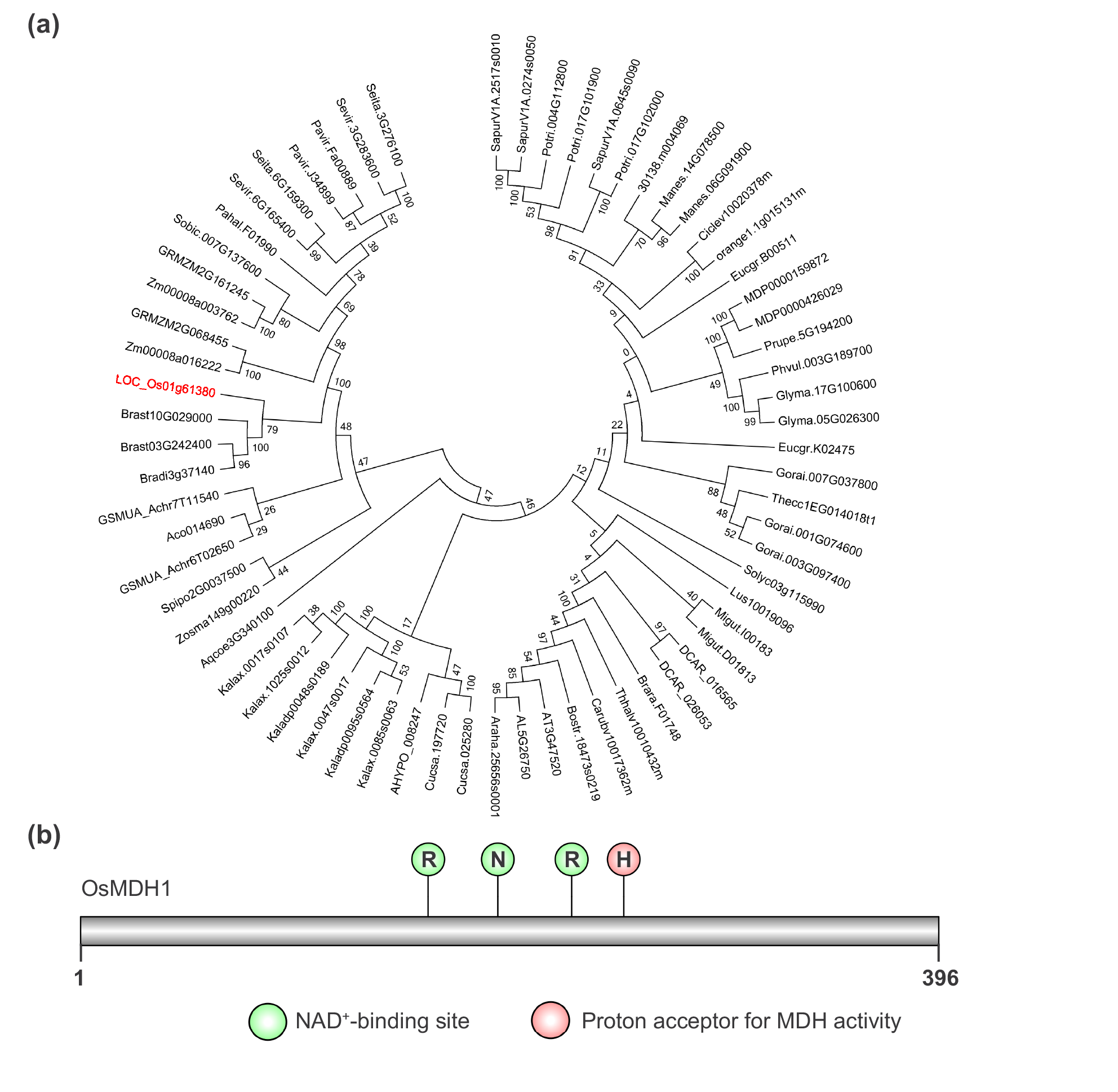

Supplement: Supplementary file 1 — Figure S1 Phylogenetic relationship of plastidial NAD‐MDH in monocot plants and catalytic sites prediction of OsMDH1. [file PBI-18-172-s003.tif]

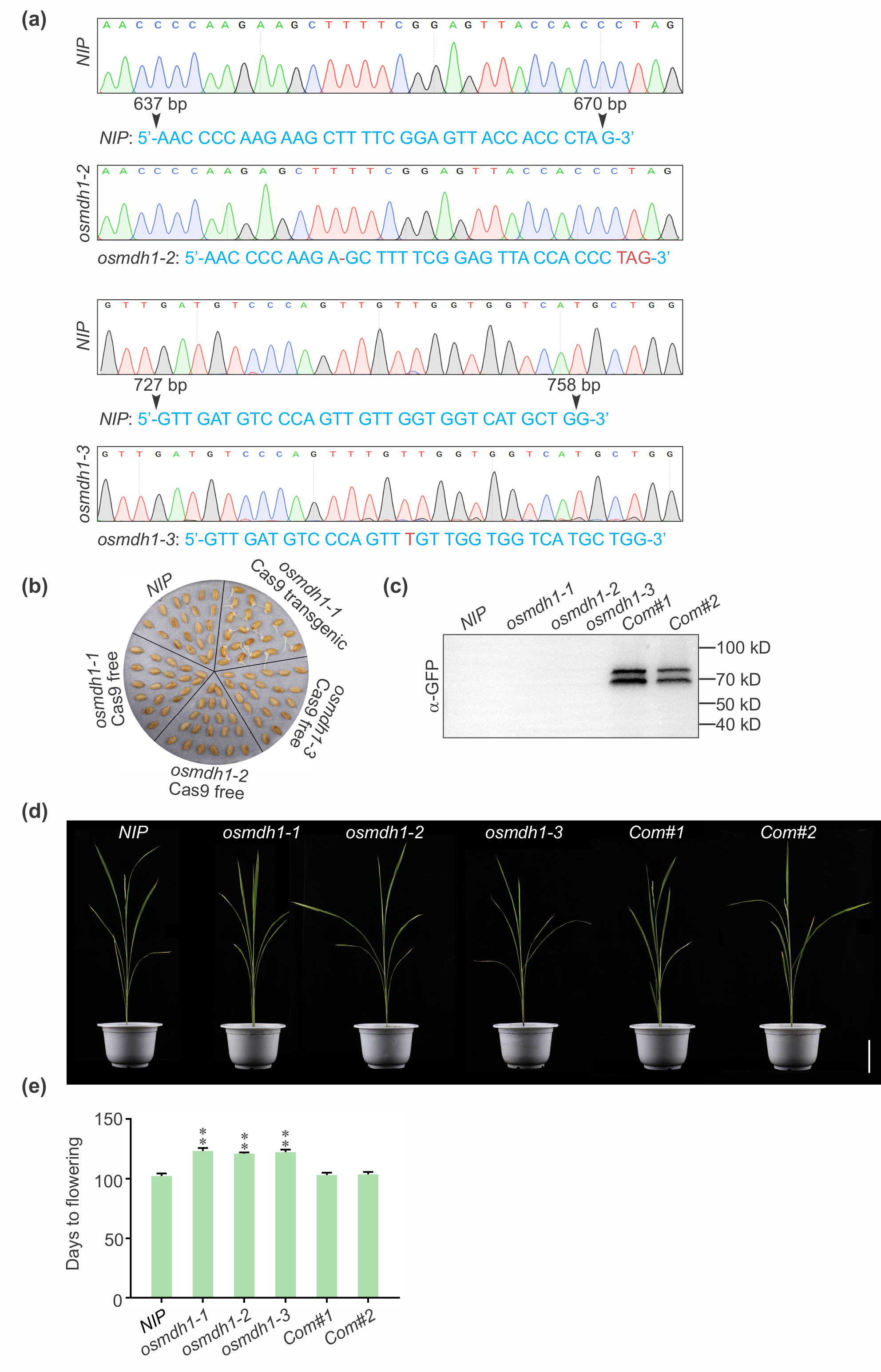

Supplement: Supplementary file 2 — Figure S2 Phenotypes of the osmdh1 mutants. [file PBI-18-172-s002.tif]

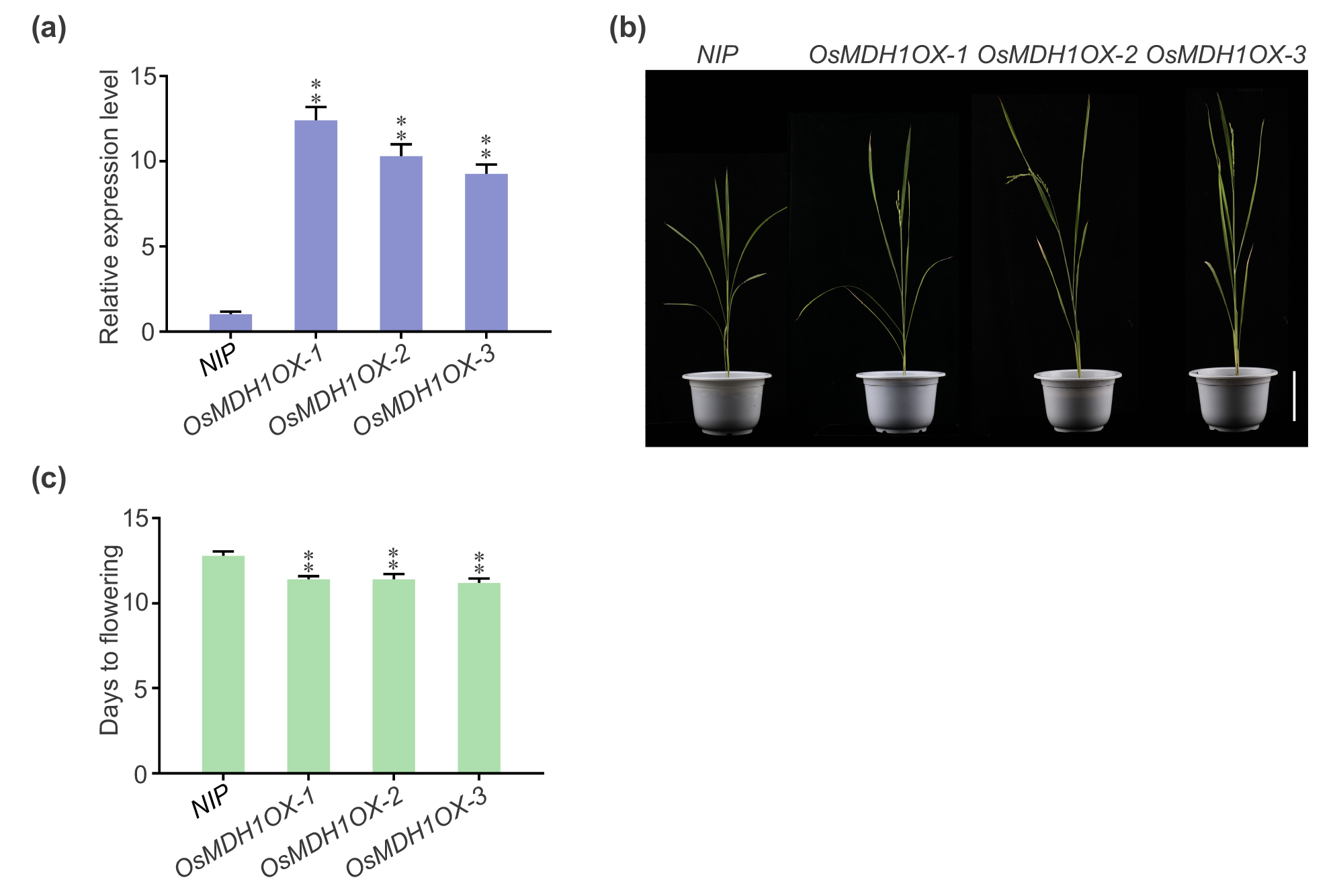

Supplement: Supplementary file 3 — Figure S3 Phenotypes of the OsMDH1OX lines. [file PBI-18-172-s005.tif]

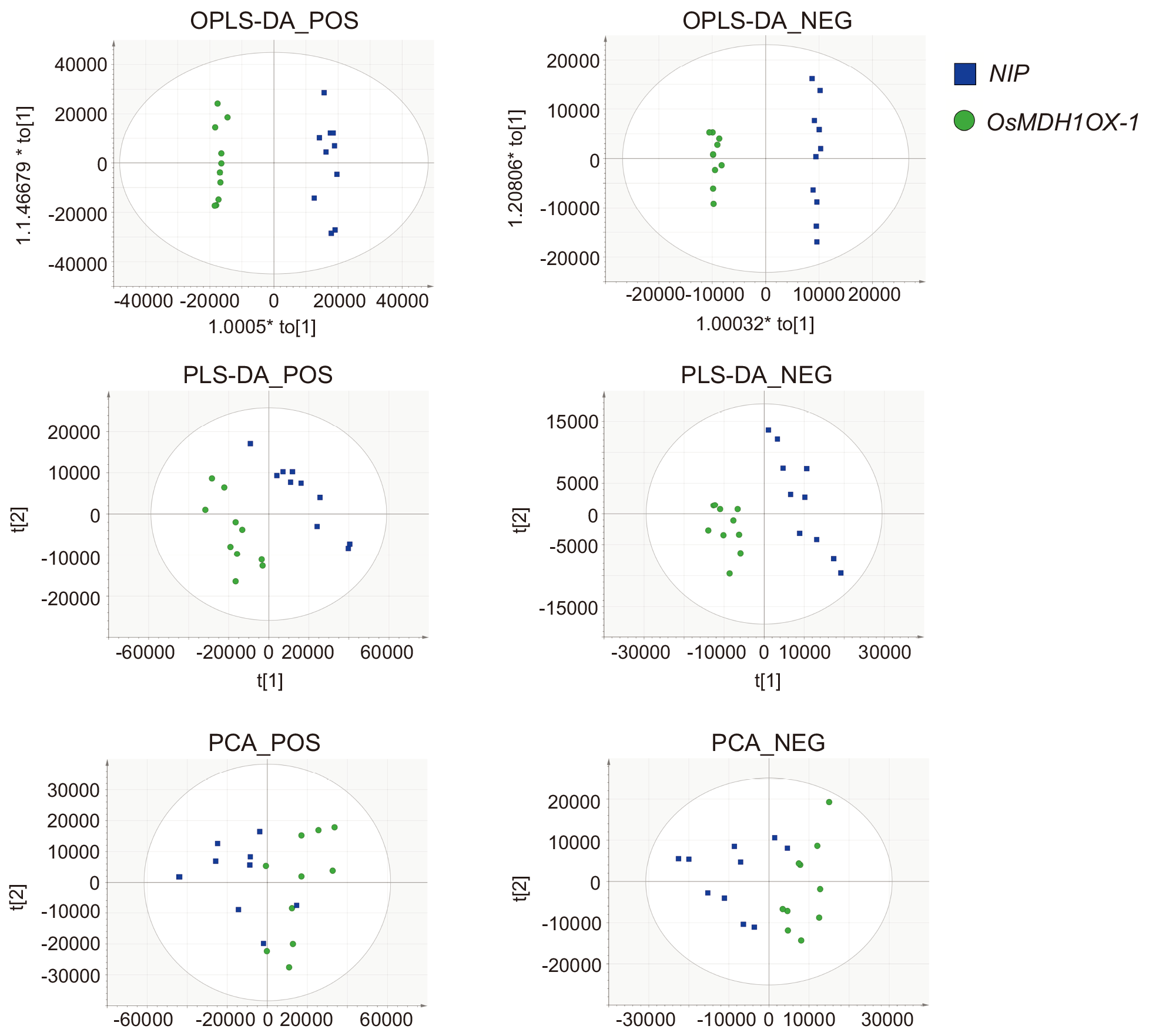

Supplement: Supplementary file 4 — Figure S4 OPLS‐DA, PLS‐DA and PCA loading plots for the discrimination between NIP vs OsMDH1OX‐1 plants under normal conditions. [file PBI-18-172-s001.tif]
